# Supplementary material for: Tissue-Specific Sex Difference in Mouse Eye and Brain Metabolome Under Fed and Fasted States
Source: Invest Ophthalmol Vis Sci. 2023 Mar 9;64(3):18. doi: 10.1167/iovs.64.3.18 (PMC10010444; doi:10.1167/iovs.64.3.18)
Supplement: Supplement 1 [file iovs-64-3-18_s001.pdf]

Supporting Information

Tissue-specific sex difference in mouse eye and brain metabolome under fed and fasted states

Meghashri Saravanan*, Rong Xu*, Olivia Roby, Yekai Wang, Siyan Zhu, Amy Lu, and Jianhai Du[&]

This file includes:

Supplementary Figure S1-6

Supplementary Table S1-17

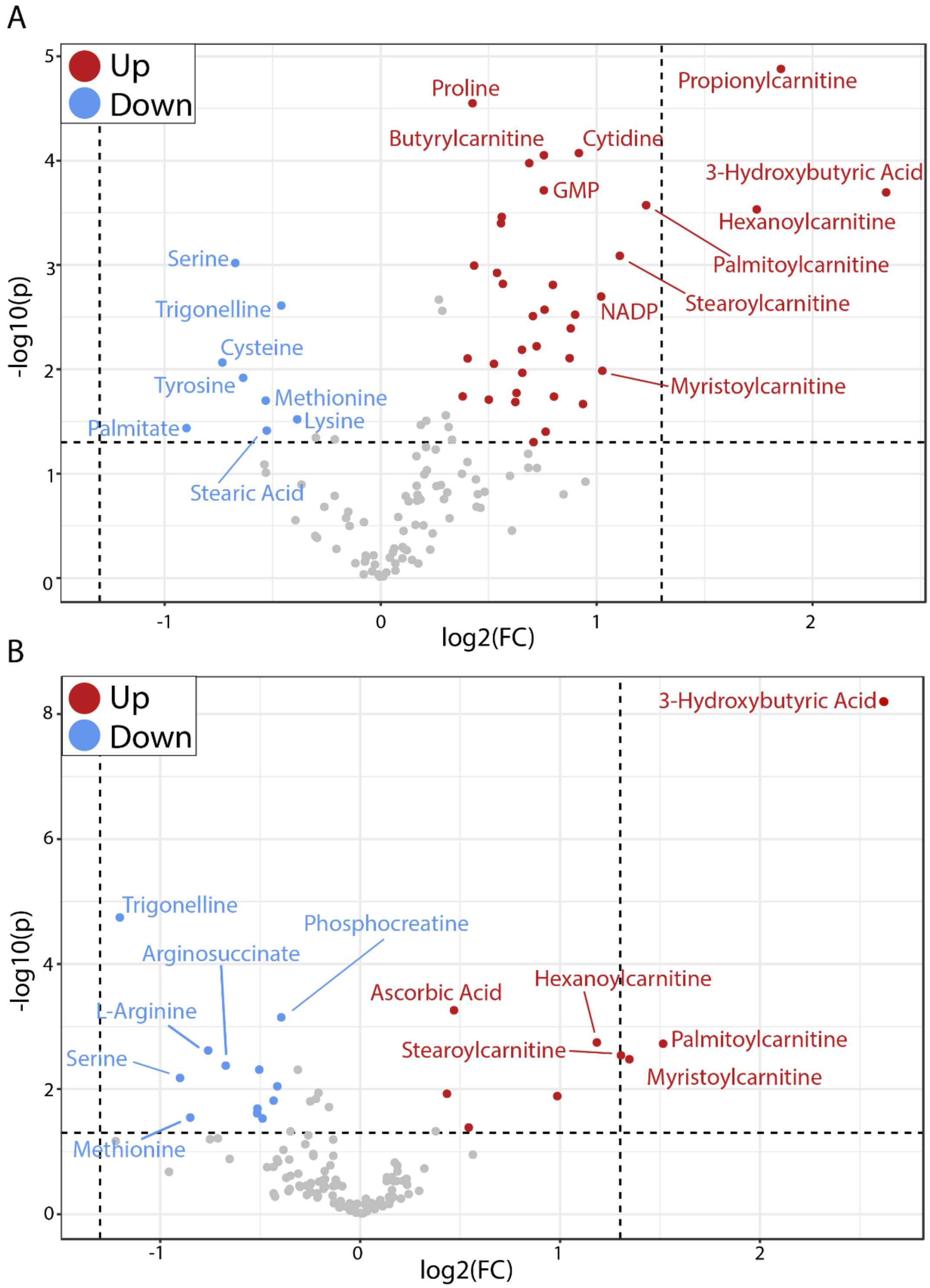

Supplemental Figure S1. (A) Volcano plots of retinal metabolites from fed vs. fasted male mice. **(B)** Volcano plots of retinal metabolites from fed vs. fasted female mice.

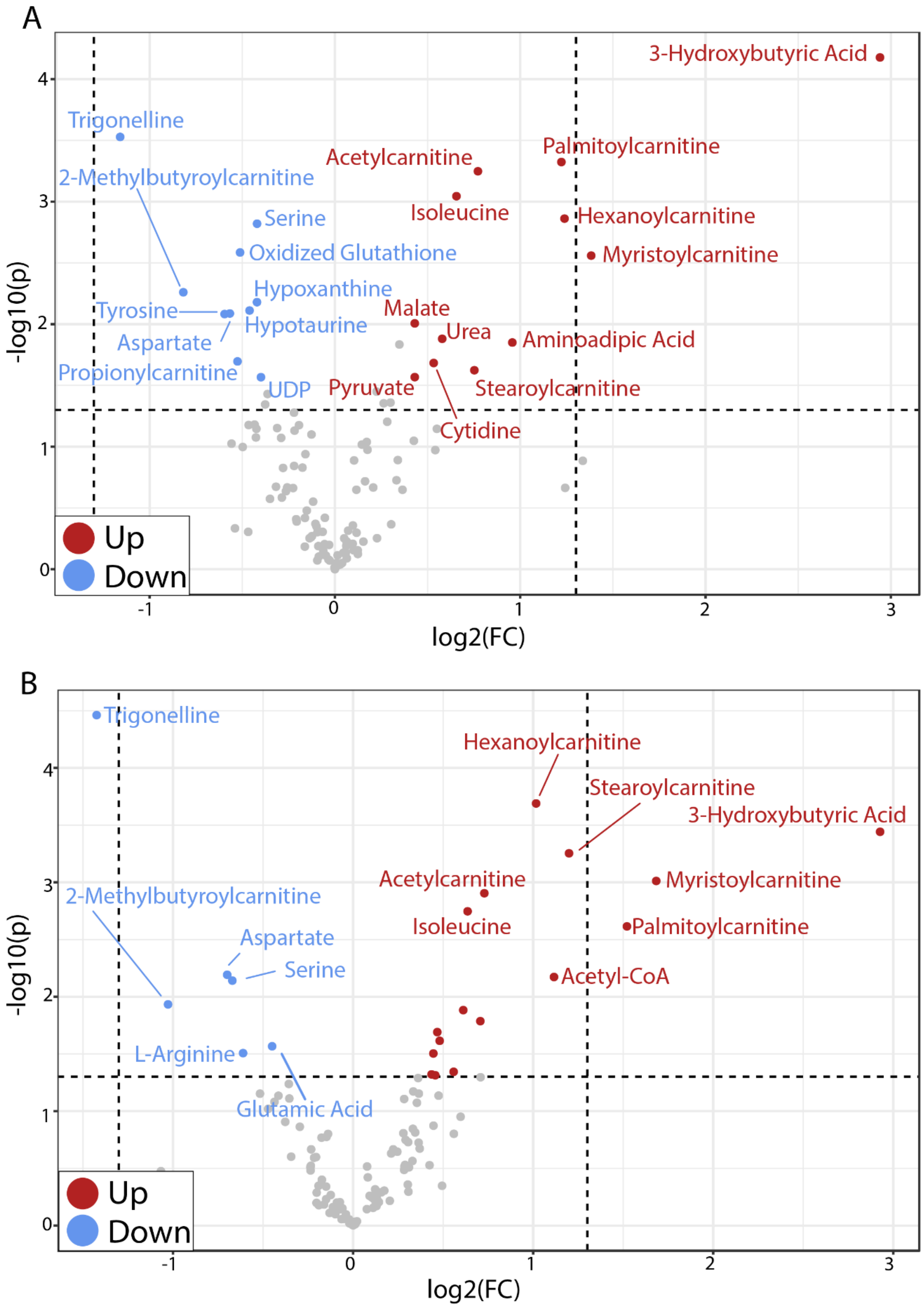

Supplemental Figure S2. (A) Volcano plots of RPE metabolites from fed vs. fasted male mice. **(B)** Volcano plots of RPE metabolites from fed and fasted male mice.

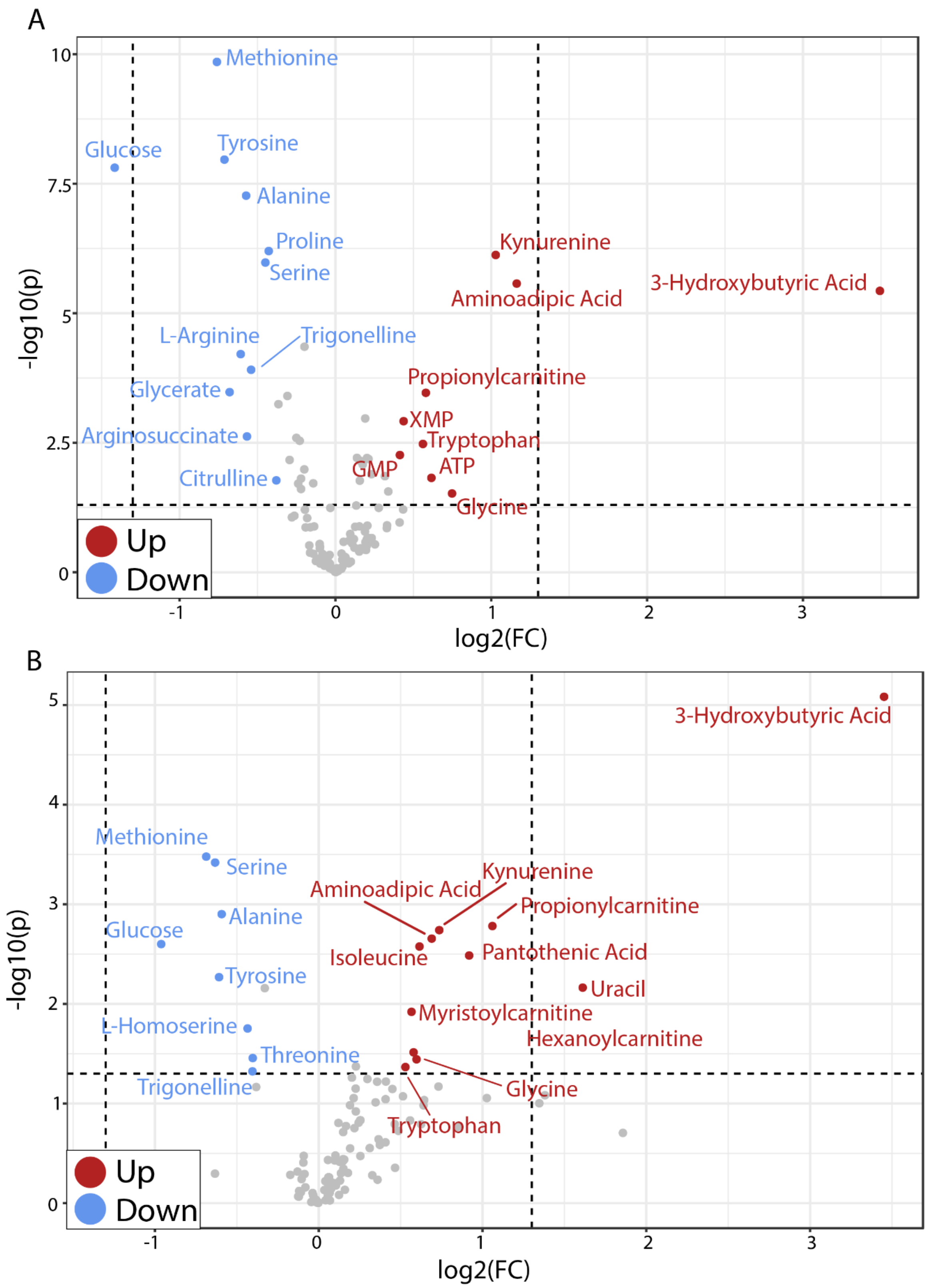

Supplemental Figure S3. (A) Volcano plots of lens metabolites from fed vs. fasted male mice. **(B)** Volcano plots of lens metabolites from fed and fasted male mice.

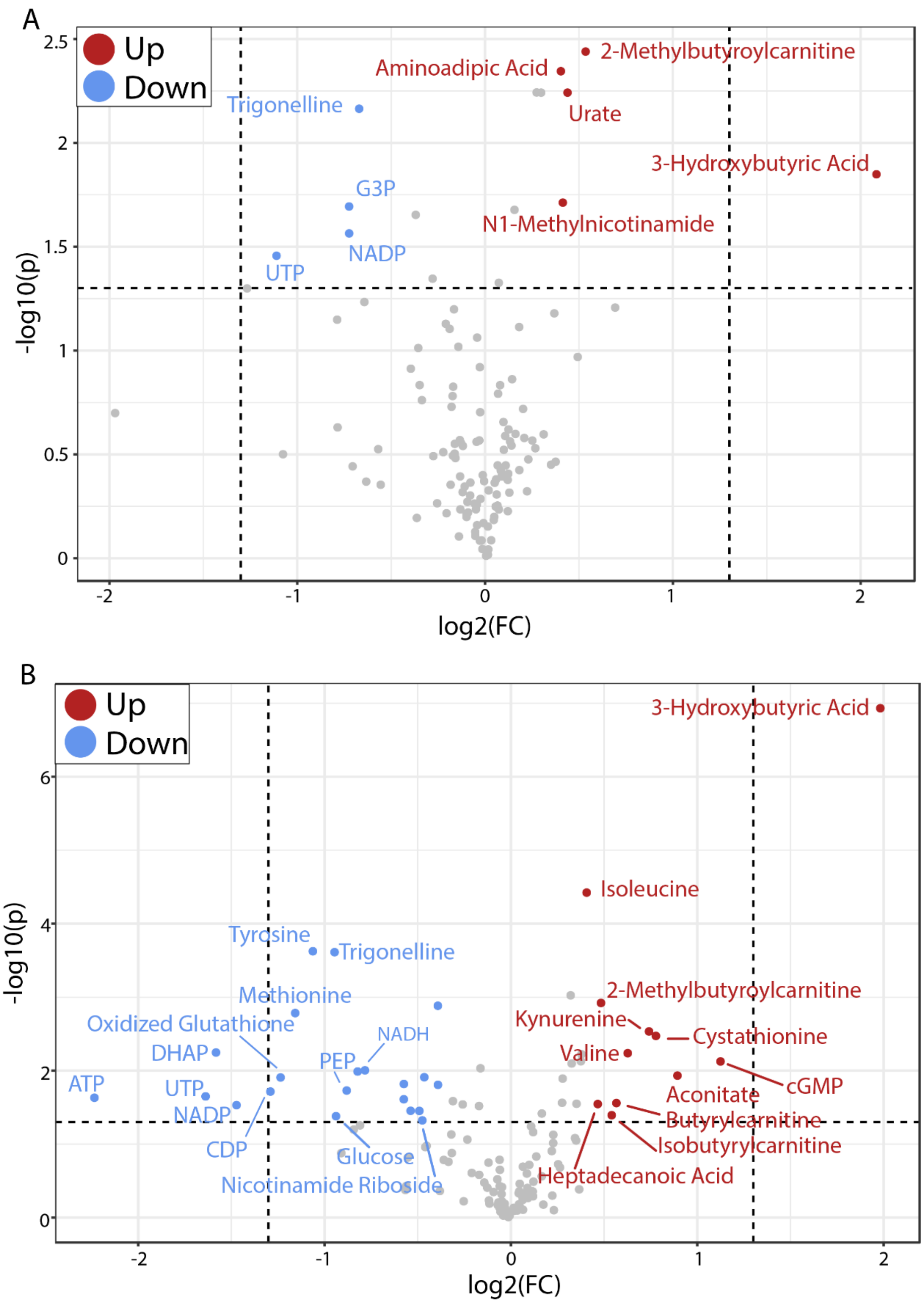

Supplemental Figure S4. (A) Volcano plots of brain metabolites from fed vs. fasted male mice. **(B)** Volcano plots of brain metabolites from fed and fasted male mice.

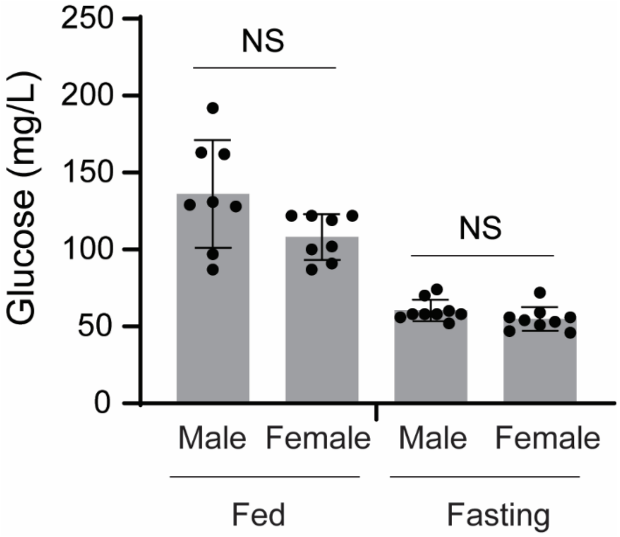

Supplemental Figure S5. Plasma glucose levels in fed and fasted mice. N=5 *P<0.05**.** Glucose levels were measured via tail bleeding using a glucometer (Contour blood glucose meter 9545C).

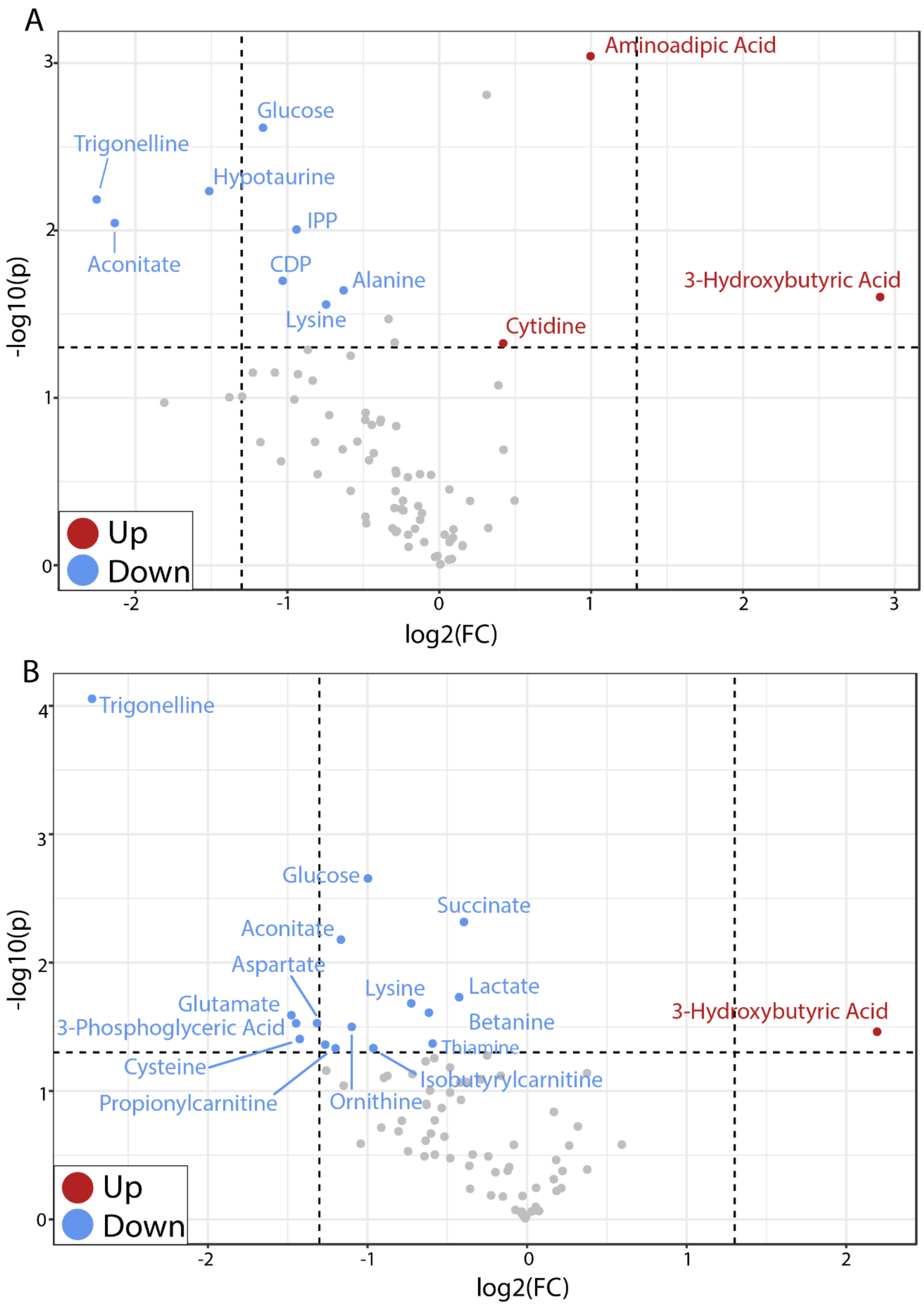

Supplemental Figure S6 (A) Volcano plots of plasma metabolites from fed vs. fasted male mice. **(B)** Volcano plots of plasma metabolites from fed and fasted male mice.

Supplemental Table S1. The lists of measured metabolites and their parameters in mass spectrometry.

Metabolite	Platform	Pathway	CAS ID
3-Hydroxybutyric acid	GCMS	Lipid/Ketone bodies	300-85-6
3-Phosphoglyceric acid	GCMS	Glycolysis	80731-10-8
a-Ketoglutarate	GCMS	TCA Cycle	328-50-7
Asparagene	GCMS	Amino Acid	70-47-3
Beta-Alanine	GCMS	Amino acid	107-95-9
Biotin	GCMS	Vitamins	58-85-5
Cholesterol	GCMS	Lipid	57-88-5
Citrate	GCMS	TCA Cycle	77-92-9
Cysteine	GCMS	Amino Acid	56-89-3
Cystine	GCMS	Amino Acid	56-89-3
DHAP	GCMS	Glycolysis	57-04-5
Fumarate	GCMS	TCA Cycle	110-17-8
Glycerate	GCMS	Glycolysis	14028-62-7
Isocitrate	GCMS	TCA Cycle	1637-73-6
Isoleucine	GCMS	Amino Acid	73-32-5
L-Histidine	GCMS	Amino Acid	332-80-9
Malate	GCMS	TCA Cycle	6915-15-7
Palmitate	GCMS	Lipid metabolism/Fatty Acids	57-10-3
PEP	GCMS	Glycolysis	138-08-9
Pyroglutamic acid	GCMS	Amino Acid	98-79-3
Pyruvate	GCMS	Glycolysis	113-24-6
Stearic acid	GCMS	Lipid metabolism/Fatty Acid	57-11-4
Taurine	GCMS	Amino Acid	107-35-7
Tryptophan	GCMS	Amino Acid	73-22-3
Uracil	GCMS	Nucleotide/Pyrimidine metabolism	66-22-8
Urea	GCMS	Urea Cycle	57-13-6
1-Methyladenosine	LCMS	Nucleotide/Purine metabolism	15763-06-1
2-Methylbutyroylcarnitine	LCMS	AcylCarnitine/Amino acid metabolism	31023-25-3
3-Aminoisobutanoic acid	LCMS	Nucleotide/pyrimidine (thymine)	144-90-1
4-Hydroxyproline	LCMS	Amino Acid metabolism/Proline/Arginine	51-35-4
AcetylCarnitine	LCMS	Lipid metabolism	14992-62-2
AcetylCholine	LCMS	Lipid/phospholipid,	51-84-3
Acetyl-CoA	LCMS	TCA Cycle/Fatty acid metabolism	72-89-9
Acetylglutamic acid	LCMS	Amino Acid metabolism/Proline/Arginine	1188-37-0
Aconitate	LCMS	TCA Cycle	499-12-7
Adenine	LCMS	Nucleotide/Purine metabolism	73-24-5
Adenosine	LCMS	Nucleotide/Purine metabolism	58-61-7
ADP	LCMS	Nucleotide/Purine metabolism	58-64-0
AICAR	LCMS	Nucleotide/Purine metabolism	3031-94-5
Alanine	LCMS	Amino Acid	56-41-7

Aminoadipic acid	LCMS	Amino Acid metabolism/Lysine	542-32-5
AMP	LCMS	Nucleotide	61-19-8
Arginosuccinate	LCMS	Urea Cycle	2387-71-5
Ascorbic acid	LCMS	Ascorbate and aldarate metabolism	50-81-7
Aspartic acid	LCMS	Amino Acid	56-84-8
ATP	LCMS	Nucleotide	987-65-5
Betaine	LCMS	Amino Acid/gly,ser, thr metabolism	590-46-5
Butyrylcarnitine	LCMS	Lipid metabolism/Fatty Acid metabolism	25576-40-3
cAMP	LCMS	Nucleotide/Purine metabolism	60-92-4
Carnitine	LCMS	Amino Acid metabolism/lys	541-15-1
Carnosine	LCMS	Amino Acids/Histidine	305-84-0
CDP	LCMS	Nucleotide/Pyrimidine metabolism	63-38-7
cGMP	LCMS	Nucleotide/Purine metabolism	7665-99-8
Choline	LCMS	Lipid metabolism	62-49-7
cis-Aconitic acid	LCMS	TCA Cycle	499-12-7
Citrulline	LCMS	Urea Cycle	372-75-8
CoA	LCMS	TCA Cycle/Fatty acid metabolism	55672-92-9
Creatine	LCMS	Amino Acids/Arg, gly	57-00-1
Creatinine	LCMS	Amino Acids/Arg, gly	60-27-5
Cystathionine	LCMS	Amino Acids/cys	56-88-2
Cytidine	LCMS	Nucleotide	65-46-3
D-2-hydroxyglutarate	LCMS	TCA cycle	103404-90-6
D-Ribulose 5-phosphate	LCMS	Glycolysis/PPP	551-85-9
Erythritol	LCMS	Sugar	149-32-6
FAD	LCMS	Nucleotide	146-14-5
G3P	LCMS	Glycolysis	142-10-9
Gamma-Aminobutyric acid	LCMS	Amino Acid metabolism/Ala, Glu, Asp	56-12-2
GDP	LCMS	Nucleotide/Purine metabolism	146-91-8
Glucose	LCMS	Glycolysis/sugar	492-62-6
Glucose 1-phosphate	LCMS	Glycolysis	59-56-3
Glucose 6-phosphate	LCMS	Glycolysis/PPP	56- 73-5
Glutamate	LCMS	Amino Acid	56-86-0
Glutamine	LCMS	Amino Acid	56-85-9
Glutathione	LCMS	Amino acid metabolism	70-18-8
Glycine	LCMS	Amino Acid	56-40-6
GMP	LCMS	Nucleotide/Purine metabolism	85-32-5
Guanine	LCMS	Nucleotide/Purine metabolism	73-40-5
Guanosine	LCMS	Nucleotide	118-00-3
Heptadecanoic acid	LCMS	Lipid metabolism/Fatty Acid metabolism	506-12-7
Hexanoylcarnitine	LCMS	Acylcarnitines/Fatty Acid metabolism	22671-29-0
Histamine	LCMS	Amino acid metabolism	51-45-6
Hypotaurine	LCMS	Taurine metabolism	300-84-5
Hypoxanthine	LCMS	Nucleotide/Purine metabolism	68-94-0

IMP	LCMS	Nucleotide/Purine metabolism	131-99-7
Inosine	LCMS	Nucleotide/Purine metabolism	58-63-9
IPP	LCMS	mevalonate pathway	18687-43-9
Isobutyrylcarnitine	LCMS	Lipid metabolism/Fatty Acid metabolism	25518-49-4
Kynurenine	LCMS	Amino Acid metabolism/Trp	343-65-7
Lactate	LCMS	Glycolysis	50-21-5
L-Arginine	LCMS	Amino Acid/Urea cycle	74-79-3
L-Asparagine	LCMS	Amino Acid/ AA metabolism/Ala, Glu, Asp	70-47-3
Leucine	LCMS	Amino Acid	61-90-5
L-Homoserine	LCMS	Amino Acid/Thr, Met, Asp	1927-25-9
Lysine	LCMS	Amino Acid	56-87-1
Methionine	LCMS	Amino Acid	63-68-3
myo-Inositol	LCMS	sugar	87-89-8
Myristoylcarnitine	LCMS	Lipid metabolism/Fatty Acid metabolism	25597-07-3
N1-Methylnicotinamide	LCMS	NAD Metabolism	3106-60-3
N-Acetyl-L-aspartic acid	LCMS	Amino Acid metabolism/Ala, Glu, Asp	997-55-7
NAD	LCMS	Nicotinate and nicotinamide metabolism	53-84-9
NADH	LCMS	Nicotinate and nicotinamide metabolism	58-68-4
NADP	LCMS	Nicotinate and nicotinamide metabolism	53-59-8
NADPH	LCMS	Nicotinate and nicotinamide metabolism	2646-71-1
Niacinamide	LCMS	Nicotinate and nicotinamide metabolism	98-92-0
Nicotinamide riboside	LCMS	Nicotinate and nicotinamide metabolism	2181-04-6
Ornithine	LCMS	Amino acid/Urea cyle	70-26-8
Oxalic acid	LCMS	Glyoxylate and dicarboxylate metabolism	144-62-7
Oxidized Glutathione	LCMS	Amino acid metabolism	13081-14-6
Palmitoylcarnitine	LCMS	Acylcarnitines/Fatty Acid metabolism	1985-18-8
Palmitoyl-CoA	LCMS	Lipid metabolism/Fatty Acid metabolism	188174-64-3
Pantothenic acid	LCMS	Vitamin/CoA	137-08-6
Phenylalanine	LCMS	Amino Acid	63-91-2
Phosphocreatine	LCMS	Amino Acid/pro and arginine	19333-65-4
Proline	LCMS	Amino Acid	4298-08-2
Propionylcarnitine	LCMS	Acylcarnitine/Fatty Acid metabolism	17298-37-2
Riboflavin	LCMS	Vitamins	83-88-5
SAM	LCMS	Amino Acid/pro and arginine	86867-01-8
Serine	LCMS	Amino Acid	56-45-1
Stearoylcarnitine	LCMS	Acylcarnitines/Fatty Acid metabolism	25597-09-5
Succinate	LCMS	TCA Cycle	110-15-6
Thiamine	LCMS	Vitamins	59-43-8
Threonine	LCMS	Amino Acid	72-19-5
Trigonelline	LCMS	Nicotinate and nicotinamide metabolism	535-83-1
Tyrosine	LCMS	Amino Acid	60-18-4
UDP	LCMS	Nucleotide/Pyrimidine metabolism	58-98-0
UDP-Glucosamine	LCMS	Nucleotide sugar	17479-04-8

Urate	LCMS	Nucleotide/Purine metabolism	69-93-2
Uridine diphosphate glucose	LCMS	Nucleotide sugar	133-89-1
UTP	LCMS	Nucleotide/pyrimidine	63-39-8
Valine	LCMS	Amino Acid	72-18-4
Xanthine	LCMS	Nucleotide	69-89-6
Xanthosine	LCMS	Nucleotide/Purine metabolism	146-80-5
XMP	LCMS	Nucleotide/Purine metabolism	523-98-8

Supplemental Table S2. Sex different retinal metabolites in different metabolic pathways in the fed state by Volcano plots.

Metabolite	Pathway	FC	log2(FC)	P Value	-log10(p)
Propionylcarnitine	Acylcarnitine/Fatty Acid metabolism	3.6136	1.8534	1.32E-05	4.8787
Proline	Amino Acid	1.343	0.42551	2.82E-05	4.5498
Cytidine	Nucleotide	1.8893	0.91789	8.47E-05	4.0723
Butyrylcarnitine	Lipid metabolism/Fatty Acid metabolism	1.6889	0.75604	8.87E-05	4.0521
Isobutyrylcarnitine	Lipid metabolism/Fatty Acid metabolism	1.6111	0.68803	0.00010577	3.9756
GMP	Nucleotide/Purine metabolism	1.6883	0.75558	0.00019268	3.7152
3-Hydroxybutyric acid	Lipid/Ketone bodies	5.0645	2.3404	0.00020165	3.6954
Palmitoylcarnitine	Acylcarnitines/Fatty Acid metabolism	2.3445	1.2293	0.00026731	3.573
Hexanoylcarnitine	Acylcarnitines/Fatty Acid metabolism	3.3436	1.7414	0.00029345	3.5325
Leucine	Amino Acid	1.4752	0.56094	0.00034592	3.461
XMP	Nucleotide/Purine metabolism	1.4717	0.55746	0.00039787	3.4003
Stearoylcarnitine	Acylcarnitines/Fatty Acid metabolism	2.1546	1.1074	0.00081669	3.0879
Serine	Amino Acid	0.6272	-0.67296	0.00095775	3.0187
Phenylalanine	Amino Acid	1.3499	0.43286	0.0010143	2.9938
Aconitate	TCA Cycle	1.4528	0.53887	0.0011904	2.9243
IMP	Nucleotide/Purine metabolism	1.4803	0.56588	0.0015179	2.8187
Pantothenic acid	Vitamin/CoA	1.7378	0.79725	0.0015518	2.8092
NADP	Nicotinate and nicotinamide metabolism	2.0291	1.0209	0.002006	2.6977
Trigonelline	Nicotinate and nicotinamide metabolism	0.727	-0.46005	0.0024475	2.6113
Oxidized glutathione	Amino acid metabolism	1.6918	0.75859	0.0026826	2.5714
CDP	Nucleotide/Pyrimidine metabolism	1.8668	0.90055	0.0029955	2.5235
N1-Methylnicotinamide	NAD Metabolism	1.6302	0.70508	0.0030881	2.5103
NADPH	Nicotinate and nicotinamide metabolism	1.8407	0.88023	0.0040566	2.3918
Pyroglutamic acid	Amino Acid	1.6497	0.7222	0.0060116	2.221
Tryptophan	Amino Acid	1.5738	0.65422	0.0064967	2.1873
GDP	Nucleotide/Purine metabolism	1.8341	0.87507	0.0078247	2.1065
G3P	Glycolysis	1.322	0.40268	0.0078706	2.104
Cysteine	Amino Acid	0.6018	-0.73268	0.008608	2.0651

UDP	Nucleotide/Pyrimidine metabolism	1.4385	0.52452	0.0088668	2.0522
Myristoylcarnitine	Lipid metabolism/Fatty Acid metabolism	2.0372	1.0266	0.010333	1.9858
Xanthine	Nucleotide	1.5758	0.65605	0.010804	1.9664
Tyrosine	Amino Acid	0.6433	-0.63648	0.01205	1.919
Nicotinamide Riboside	Nicotinate and nicotinamide metabolism	1.5466	0.62907	0.016849	1.7734
D-2-hydroxyglutarate	TCA cycle	1.3012	0.37988	0.018187	1.7402
UTP	Nucleotide/pyrimidine	1.7443	0.80261	0.018249	1.7388
ADP	Nucleotide/Purine metabolism	1.4149	0.50071	0.019531	1.7093
Methionine	Amino Acid	0.6916	-0.53208	0.019937	1.7003
FAD	Nucleotide	1.541	0.62388	0.020585	1.6864
Palmitoyl-CoA	Lipid metabolism/Fatty Acid metabolism	1.9143	0.93683	0.021527	1.667
Lysine	Amino Acid	0.7653	-0.38599	0.030136	1.5209
Palmitate	Lipid metabolism/Fatty Acids	0.5363	-0.89884	0.036594	1.4366
Stearic acid	Lipid metabolism/Fatty Acid	0.6938	-0.5274	0.038629	1.4131
Kynurenine	Amino Acid metabolism/Trp	1.6981	0.76389	0.039618	1.4021
Guanine	Nucleotide/Purine metabolism	1.6336	0.70809	0.049879	1.3021

Supplemental Table S3. Changed retinal metabolites in different metabolic pathways in fed vs. fasted male mice by Volcano plots.

Metabolite	Pathway	FC	log2(FC)	P Value	-log10(p)
Propionylcarnitine	Acylcarnitine/Fatty Acid metabolism	3.6136	1.8534	1.32E-05	4.8787
Proline	Amino Acid	1.343	0.42551	2.82E-05	4.5498
Cytidine	Nucleotide	1.8893	0.91789	8.47E-05	4.0723
Butyrylcarnitine	Lipid metabolism/Fatty Acid metabolism	1.6889	0.75604	8.87E-05	4.0521
Isobutyrylcarnitine	Lipid metabolism/Fatty Acid metabolism	1.6111	0.68803	0.00010577	3.9756
GMP	Nucleotide/Purine metabolism	1.6883	0.75558	0.00019268	3.7152
3-Hydroxybutyric acid	Lipid/Ketone bodies	5.0645	2.3404	0.00020165	3.6954
Palmitoylcarnitine	Acylcarnitines/Fatty Acid metabolism	2.3445	1.2293	0.00026731	3.573
Hexanoylcarnitine	Acylcarnitines/Fatty Acid metabolism	3.3436	1.7414	0.00029345	3.5325
Leucine	Amino Acid	1.4752	0.56094	0.00034592	3.461
XMP	Nucleotide/Purine metabolism	1.4717	0.55746	0.00039787	3.4003
Stearoylcarnitine	Acylcarnitines/Fatty Acid metabolism	2.1546	1.1074	0.00081669	3.0879
Serine	Amino Acid	0.62722	-0.67296	0.00095775	3.0187
Phenylalanine	Amino Acid	1.3499	0.43286	0.0010143	2.9938
Aconitate	TCA Cycle	1.4528	0.53887	0.0011904	2.9243
IMP	Nucleotide/Purine metabolism	1.4803	0.56588	0.0015179	2.8187
Pantothenic acid	Vitamin/CoA	1.7378	0.79725	0.0015518	2.8092
NADP	Nicotinate and nicotinamide metabolism	2.0291	1.0209	0.002006	2.6977
Trigonelline	Nicotinate and nicotinamide metabolism	0.72696	-0.46005	0.0024475	2.6113
Oxidized glutathione	Amino acid metabolism	1.6918	0.75859	0.0026826	2.5714
CDP	Nucleotide/Pyrimidine metabolism	1.8668	0.90055	0.0029955	2.5235
N1-Methylnicotinamide	NAD Metabolism	1.6302	0.70508	0.0030881	2.5103
NADPH	Nicotinate and nicotinamide metabolism	1.8407	0.88023	0.0040566	2.3918
Pyroglutamic acid	Amino Acid	1.6497	0.7222	0.0060116	2.221
Tryptophan	Amino Acid	1.5738	0.65422	0.0064967	2.1873
GDP	Nucleotide/Purine metabolism	1.8341	0.87507	0.0078247	2.1065
G3P	Glycolysis	1.322	0.40268	0.0078706	2.104
Cysteine	Amino Acid	0.60178	-0.73268	0.008608	2.0651

UDP	Nucleotide/Pyrimidine metabolism	1.4385	0.52452	0.0088668	2.0522
Myristoylcarnitine	Lipid metabolism/Fatty Acid metabolism	2.0372	1.0266	0.010333	1.9858
Xanthine	Nucleotide	1.5758	0.65605	0.010804	1.9664
Tyrosine	Amino Acid	0.64328	-0.63648	0.01205	1.919
Nicotinamide Riboside	Nicotinate and nicotinamide metabolism	1.5466	0.62907	0.016849	1.7734
D-2-hydroxyglutarate	TCA cycle	1.3012	0.37988	0.018187	1.7402
UTP	Nucleotide/pyrimidine	1.7443	0.80261	0.018249	1.7388
ADP	Nucleotide/Purine metabolism	1.4149	0.50071	0.019531	1.7093
Methionine	Amino Acid	0.69156	-0.53208	0.019937	1.7003
FAD	Nucleotide	1.541	0.62388	0.020585	1.6864
Palmitoyl-CoA	Lipid metabolism/Fatty Acid metabolism	1.9143	0.93683	0.021527	1.667
Lysine	Amino Acid	0.76526	-0.38599	0.030136	1.5209
Palmitate	Lipid metabolism/Fatty Acids	0.53632	-0.89884	0.036594	1.4366
Stearic acid	Lipid metabolism/Fatty Acid	0.6938	-0.5274	0.038629	1.4131
Kynurenine	Amino Acid metabolism/Trp	1.6981	0.76389	0.039618	1.4021
Guanine	Nucleotide/Purine metabolism	1.6336	0.70809	0.049879	1.3021

Supplemental Table S4. Changed retinal metabolites in different metabolic pathways in fed vs. fasted female mice by Volcano plots.

Metabolite	Pathway	FC	log2(FC)	P Value	-log10(p)
3-Hydroxybutyric acid	Lipid/Ketone bodies	6.1512	2.6209	6.38E-09	8.1955
Trigonelline	Nicotinate and nicotinamide metabolism	0.43467	-1.202	1.80E-05	4.7458
Ascorbic acid	Ascorbate and aldarate metabolism	1.3844	0.46922	0.00054799	3.2612
Phosphocreatine	Amino Acid/Pro and Arg	0.76063	-0.39473	0.00071183	3.1476
Hexanoylcarnitine	Acylcarnitines/Fatty Acid metabolism	2.2721	1.184	0.0017945	2.7461
Palmitoylcarnitine	Acylcarnitines/Fatty Acid metabolism	2.8586	1.5153	0.0018785	2.7262
L-Arginine	Amino Acid/Urea cycle	0.5904	-0.76024	0.0024084	2.6183
Stearoylcarnitine	Acylcarnitines/Fatty Acid metabolism	2.4699	1.3044	0.0028844	2.5399
Myristoylcarnitine	Lipid metabolism/Fatty Acid metabolism	2.5431	1.3466	0.0033302	2.4775
Arginosuccinate	Urea Cycle	0.62751	-0.67229	0.0042092	2.3758
Aspartic acid	Amino Acid	0.70485	-0.50461	0.0048872	2.3109
Serine	Amino Acid	0.53553	-0.90095	0.0066322	2.1783
Alanine	Amino Acid	0.75038	-0.41431	0.0090249	2.0446
Pantothenic acid	Vitamin/CoA	1.3511	0.43416	0.01189	1.9248
Propionylcarnitine	Acylcarnitine/Fatty Acid metabolism	1.9806	0.98592	0.012951	1.8877
Carnitine	Amino Acid metabolism/Lys	0.74072	-0.43301	0.015299	1.8153
Aminoadipic acid	Amino Acid metabolism/Ly	0.70052	-0.51351	0.020473	1.6888
Threonine	Amino Acid	0.69972	-0.51514	0.024436	1.612
Methionine	Amino Acid	0.55488	-0.84975	0.028497	1.5452
L-Homoserine	Amino Acid/Thr, Met, Asp	0.71292	-0.48818	0.029511	1.53
Kynurenine	Amino Acid metabolism/Trp	1.457	0.54301	0.041153	1.3856

Supplemental Table S5. Changed RPE metabolites in different metabolic pathways in the fed state by Volcano plots.

Metabolite	Pathway	FC	log2(FC)	P Value	-log10(p)
Succinate	TCA Cycle	0.28256	-1.8233	5.76E-06	5.2395
Beta-Alanine	Amino Acid	0.50734	-0.97897	0.0001627	3.7887
Taurine	Amino Acid	0.61686	-0.69698	0.0026604	2.5751
4-Hydroxyproline	Amino Acid metabolism/Pro, Arg	0.61081	-0.71121	0.0028086	2.5515
Acetyl-CoA	TCA Cycle/Fatty acid metabolism	0.58175	-0.78152	0.0046309	2.3343
AcetylCholine	Lipid/phospholipid,	0.30612	-1.7078	0.0075427	2.1225
Hypotaurine	Taurine metabolism	0.51827	-0.94823	0.0078318	2.1061
Hypoxanthine	Nucleotide/Purine metabolism	0.71865	-0.47664	0.011852	1.9262
ATP	Nucleotide	0.50062	-0.9982	0.012307	1.9098
NAD	Nicotinate and nicotinamide metabolism	0.69381	-0.52739	0.015164	1.8192
Citrate	TCA Cycle	0.70483	-0.50466	0.018697	1.7282
Creatinine	Amino Acids/Arg, gly	0.56817	-0.8156	0.022438	1.649
Pantothenic acid	Vitamin/CoA	1.4528	0.53888	0.026994	1.5687
Histamine	Amino acid metabolism	0.66588	-0.58666	0.028587	1.5438
Guanine	Nucleotide/Purine metabolism	0.66912	-0.57967	0.031756	1.4982
Palmitoylcarnitine	Acylcarnitines/Fatty Acid metabolism	0.72661	-0.46075	0.032625	1.4865
Propionylcarnitine	Acylcarnitine/Fatty Acid metabolism	0.72521	-0.46353	0.034629	1.4606
D-Ribulose 5-phosphate	Glycolysis/PPP	0.67922	-0.55806	0.040824	1.3891
Carnosine	Amino Acids/Histidine	0.57018	-0.81052	0.048639	1.313

Supplemental Table S6. Changed RPE metabolites in different metabolic pathways in fed vs. fasted male mice by Volcano plots.

**Metabolite**	**Pathway**	**FC**	**log2(FC)**	**P Value**	**-log10(p)**
3-Hydroxybutyric acid	Lipid/Ketone bodies	7.6816	2.9414	6.65E-05	4.1774
Trigonelline	Nicotinate and nicotinamide metabolism	0.44784	-1.1589	0.0002962	3.5284
Palmitoylcarnitine	Acylcarnitines/Fatty Acid metabolism	2.333	1.2222	0.0004746	3.3237
AcetylCarnitine	Lipid/phospholipid	1.7073	0.7717	0.0005647	3.2482
Isoleucine	Amino Acid	1.5756	0.65586	0.000902	3.0448
Hexanoylcarnitine	Acylcarnitines/Fatty Acid metabolism	2.3603	1.239	0.0013745	2.8618
Serine	Amino Acid	0.74735	-0.42013	0.0015128	2.8202
Oxidized glutathione	Amino acid metabolism	0.70137	-0.51175	0.002598	2.5854
Myristoylcarnitine	Lipid metabolism/Fatty Acid metabolism	2.608	1.383	0.0027566	2.5596
2-Methylbutyroylcarnitine	AcylCarnitine/Amino acid metabolism	0.56719	-0.8181	0.005489	2.2605
Hypoxanthine	Nucleotide/Purine metabolism	0.74717	-0.42049	0.0066116	2.1797
Hypotaurine	Taurine metabolism	0.72661	-0.46076	0.0077346	2.1116
Aspartic acid	Amino Acid	0.675	-0.56704	0.0081778	2.0874
Tyrosine	Amino Acid	0.66191	-0.5953	0.0082668	2.0827
Malate	TCA Cycle	1.348	0.43078	0.0098496	2.0066
Urea	Urea Cycle	1.4937	0.57887	0.013164	1.8806
Aminoadipic acid	Amino Acid metabolism/Lysine	1.9418	0.95738	0.014115	1.8503
Propionylcarnitine	Acylcarnitine/Fatty Acid metabolism	0.69463	-0.52568	0.020135	1.696
Cytidine	Nucleotide	1.4469	0.53297	0.020744	1.6831
Stearoylcarnitine	Acylcarnitines/Fatty Acid metabolism	1.6847	0.75249	0.023764	1.6241
Pyruvate	Glycolysis	1.3479	0.43076	0.027049	1.5678
UDP	Nucleotide/Pyrimidine metabolism	0.75831	-0.39913	0.027087	1.5672

Supplemental Table S7. Changed RPE metabolites in different metabolic pathways in fed vs. fasted female mice by Volcano plots.

Metabolite	Pathway	FC	log2(FC)	P Value	-log10(p)
Trigonelline	Nicotinate and nicotinamide metabolism	0.37264	-1.4241	3.46E-05	4.4613
Hexanoylcarnitine	Acylcarnitines/Fatty Acid metabolism	2.023	1.0165	0.0002045	3.6892
3-Hydroxybutyric acid	Lipid/Ketone bodies	7.6035	2.9267	0.0003618	3.4415
Stearoylcarnitine	Acylcarnitines/Fatty Acid metabolism	2.2963	1.1993	0.0005578	3.2535
Myristoylcarnitine	Lipid metabolism/Fatty Acid metabolism	3.2121	1.6835	0.0009731	3.0119
AcetylCarnitine	Lipid metabolism	1.6574	0.72896	0.0012461	2.9044
Isoleucine	Amino Acid	1.554	0.63598	0.0017928	2.7465
Palmitoylcarnitine	Acylcarnitines/Fatty Acid metabolism	2.8697	1.5209	0.002428	2.6147
Aspartic acid	Amino Acid	0.61598	-0.69905	0.0064289	2.1919
Acetyl-CoA	TCA Cycle/Fatty acid metabolism	2.1671	1.1158	0.006723	2.1724
Serine	Amino Acid	0.62834	-0.67038	0.0072295	2.1409
2-Methylbutyroylcarnitine	AcylCarnitine/Amino acid metabolism	0.49054	-1.0276	0.011665	1.9331
Isocitrate	TCA Cycle	1.5283	0.6119	0.013093	1.883
Citrate	TCA Cycle	1.632	0.70663	0.016346	1.7866
Malate	TCA Cycle	1.3829	0.46766	0.02038	1.6908
Creatinine	Amino Acids/Arg, gly	1.3956	0.4809	0.024285	1.6147
Glutamic acid	Amino Acid	0.73231	-0.44947	0.027103	1.567
L-Arginine	Amino Acid/Urea cycle	0.65482	-0.61083	0.03111	1.5071
Phosphocreatine	Amino Acid/pro and arginine	1.3621	0.44585	0.031335	1.504
Carnosine	Amino Acids/Histidine	1.4727	0.55848	0.045295	1.3439
Pantothenic acid	Vitamin/CoA	1.3527	0.43579	0.047715	1.3213
Cytidine	Nucleotide	1.3723	0.45659	0.048568	1.3136

Supplemental Table S8. Changed lens metabolites in different metabolic pathways in the fed state by Volcano plots.

Metabolite	Pathway	FC	log2(FC)	P Value	-log10(p)
4-Hydroxyproline	Amino Acid metabolism/Pro, Arg	0.42469	-1.2355	1.69E-07	6.7712
Hypotaurine	Taurine metabolism	0.5417	-0.88443	1.61E-05	4.7944
Ascorbic acid	Ascorbate and aldarate metabolism	0.12633	-2.9847	3.80E-05	4.4204
Glutathione	Amino acid metabolism	0.25927	-1.9475	8.57E-05	4.0668
UDP-Glucosamine	Nucleotide sugar	0.4611	-1.1169	0.000487	3.3125
Urate	Nucleotide/Purine metabolism	0.55914	-0.83872	0.0006381	3.1951
IMP	Nucleotide/Purine metabolism	0.68324	-0.54953	0.0007074	3.1503
Propionylcarnitine	Acylcarnitine/Fatty Acid metabolism	0.5577	-0.84245	0.000992	3.0035
NADH	Nicotinate and nicotinamide metabolism	0.4921	-1.023	0.001009	2.9961
AMP	Nucleotide	0.67795	-0.56074	0.0011322	2.9461
UDP	Nucleotide/ Pyrimidine metabolism	0.33224	-1.5897	0.0017076	2.7676
CDP	Nucleotide/ Pyrimidine metabolism	0.3354	-1.576	0.001726	2.7629
Kynurenine	Amino Acid metabolism/Trp	1.5	0.58496	0.0021433	2.6689
D-Ribulose 5-phosphate	Glycolysis/PPP	0.697	-0.52077	0.0023475	2.6294
ADP	Nucleotide/Purine metabolism	0.41638	-1.264	0.0025508	2.5933
GDP	Nucleotide/Purine metabolism	0.37867	-1.401	0.0041162	2.3855
IPP	mevalonate pathway	0.67784	-0.56097	0.0046131	2.336
Butyrylcarnitine	Lipid metabolism/Fatty Acid metabolism	0.68725	-0.54109	0.0051714	2.2864
N-Acetyl-L-aspartic acid	Amino Acid metabolism/Ala, Glu, Asp	0.65407	-0.61249	0.0053861	2.2687
Isobutyrylcarnitine	Lipid metabolism/Fatty Acid metabolism	0.70134	-0.51182	0.0063538	2.197
Cystine	Amino Acid	1.6243	0.69978	0.0066549	2.1769
Hexanoylcarnitine	Acylcarnitines/Fatty Acid metabolism	0.6721	-0.57325	0.0069427	2.1585
Palmitoylcarnitine	Acylcarnitines/Fatty Acid metabolism	0.73883	-0.43669	0.0073081	2.1362

Erythritol	Sugar	0.76338	-0.38952	0.0092814	2.0324
2-Methylbutyroylcarnitine	AcylCarnitine/Amino acid metabolism	0.72039	-0.47316	0.0099399	2.0026
Phosphocreatine	Amino Acid/pro and arginine	0.49136	-1.0251	0.010897	1.9627
Myristoylcarnitine	Lipid metabolism/Fatty Acid metabolism	0.61277	-0.70658	0.012593	1.8999
Glucose	Glycolysis/sugar	1.4773	0.56298	0.012673	1.8971
Acetylglutamic acid	Amino Acid metabolism/Proline/Arginine	0.76847	-0.37993	0.012866	1.8906
Nicotinamide Riboside	Nicotinate and nicotinamide metabolism	0.66388	-0.59102	0.013104	1.8826
GMP	Nucleotide/Purine metabolism	0.629	-0.66887	0.013846	1.8587
Glucose 6-phosphate	Glycolysis/PPP	0.71809	-0.47776	0.020177	1.6951
AcetylCholine	Lipid/phospholipid, ligang	0.61312	-0.70577	0.024482	1.6111
Adenine	Nucleotide/Purine metabolism	2.2215	1.1515	0.025506	1.5934
Fumarate	TCA Cycle	0.62599	-0.67578	0.025734	1.5895
Arginosuccinate	Urea Cycle	0.67647	-0.5639	0.028288	1.5484
UTP	Nucleotide/pyrimidine	0.48947	-1.0307	0.03114	1.5067
Aconitate	TCA Cycle	0.61004	-0.71301	0.032692	1.4856
Uracil	Nucleotide/ Pyrimidine metabolism	0.73613	-0.44196	0.033105	1.4801
Tryptophan	Amino Acid	1.3055	0.38462	0.036312	1.44
Isocitrate	TCA Cycle	0.56684	-0.81899	0.037813	1.4224
Malate	TCA Cycle	0.67148	-0.57457	0.039126	1.4075
Citrate	TCA Cycle	0.6029	-0.73002	0.041385	1.3832
a-Ketoglutarate	TCA Cycle	0.60481	-0.72544	0.042366	1.373
3-Phosphoglyceric acid	Glycolysis	0.55339	-0.85364	0.048102	1.3178

Supplemental Table S9. Changed lens metabolites in different metabolic pathways in fed vs. fasted male mice by Volcano plots.

Metabolite	Pathway	FC	log2(FC)	P Value	-log10(p)
4-Hydroxyproline	Amino Acid metabolism/Proline/Arginine	0.45368	-1.1403	1.06E-07	6.9736
NADH	Nicotinate and nicotinamide metabolism	0.52409	-0.9321	5.55E-07	6.2555
ADP	Nucleotide/Purine metabolism	0.37578	-1.412	6.89E-07	6.1619
UDP	Nucleotide/Pyrimidine metabolism	0.29544	-1.7591	3.26E-06	5.4863
CDP	Nucleotide/Pyrimidine metabolism	0.26815	-1.8989	3.86E-06	5.4129
IMP	Nucleotide/Purine metabolism	0.70389	-0.50658	6.97E-06	5.1568
AMP	Nucleotide	0.68836	-0.53877	8.22E-06	5.0853
Glutathione	Amino acid metabolism	0.28772	-1.7973	1.61E-05	4.7922
GDP	Nucleotide/Purine metabolism	0.3004	-1.735	2.40E-05	4.6207
Ascorbic acid	Ascorbate and aldarate metabolism	0.13111	-2.9312	3.13E-05	4.5041
Hypotaurine	Taurine metabolism	0.6706	-0.57648	6.82E-05	4.1663
GMP	Nucleotide/Purine metabolism	0.64486	-0.63293	9.42E-05	4.026
ATP	Nucleotide	0.40133	-1.3171	0.00024	3.6194
Adenine	Nucleotide/Purine metabolism	2.2561	1.1739	0.00043	3.3668
IPP	mevalonate pathway	0.74224	-0.43004	0.000651	3.1861
Aminoadipic acid	Amino Acid metabolism/Lysine	0.63879	-0.64658	0.000893	3.0492
XMP	Nucleotide/Purine metabolism	0.6833	-0.54941	0.001574	2.803
Glucose	Glycolysis/sugar	2.0243	1.0174	0.00194	2.7122
Nicotinamide riboside	Nicotinate and nicotinamide metabolism	0.71199	-0.49007	0.002375	2.6244
Pantothenic acid	Vitamin/CoA	1.5388	0.62182	0.003703	2.4314
Thiamine	Vitamins	0.44959	-1.1533	0.003934	2.4052
Cystine	Amino Acid	2.3494	1.2323	0.004484	2.3484
3-Phosphoglyceric acid	Glycolysis	0.61357	-0.7047	0.009814	2.0082
Glycerate	Glycolysis	2.0418	1.0298	0.015175	1.8189
UTP	Nucleotide/pyrimidine	0.5478	-0.86828	0.015874	1.7993
UDP-Glucosamine	Nucleotide sugar	0.72676	-0.46045	0.016041	1.7948
Uracil	Nucleotide/Pyrimidine metabolism	1.9515	0.96461	0.016134	1.7923
NAD	Nicotinate and nicotinamide metabolism	0.6555	-0.60933	0.028197	1.5498

Supplemental Table S10. Changed lens metabolites in different metabolic pathways in fed vs. fasted female mice by Volcano plots.

Metabolite	Pathway	FC	log2(FC)	P Value	-log10(p)
Methionine	Amino Acid	0.59031	-0.76045	1.41E-10	9.8516
Tyrosine	Amino Acid	0.61037	-0.71224	1.08E-08	7.9659
Glucose	Glycolysis/sugar	0.3747	-1.4162	1.55E-08	7.81
Alanine	Amino Acid	0.67223	-0.57297	5.37E-08	7.2701
Proline	Amino Acid	0.74338	-0.42782	6.30E-07	6.201
Kynurenine	Amino Acid metabolism/Trp	2.04	1.0286	7.50E-07	6.1249
Serine	Amino Acid	0.73233	-0.44943	1.05E-06	5.9786
Aminoadipic acid	Amino Acid metabolism/Lysine	2.2399	1.1634	2.67E-06	5.5729
3-Hydroxybutyric acid	Lipid/Ketone bodies	11.264	3.4937	3.70E-06	5.4319
L-Arginine	Amino Acid/Urea cycle	0.65624	-0.60771	6.15E-05	4.211
Trigonelline	Nicotinate and nicotinamide metabolism	0.68732	-0.54095	0.0001234	3.9088
Glycerate	Glycolysis	0.62468	-0.6788	0.0003331	3.4774
Propionylcarnitine	Acylcarnitine/Fatty Acid metabolism	1.4952	0.58031	0.0003437	3.4638
XMP	Nucleotide/Purine metabolism	1.3544	0.43763	0.001212	2.9165
Arginosuccinate	Urea Cycle	0.67479	-0.56749	0.0023894	2.6217
Tryptophan	Amino Acid	1.4758	0.56152	0.0033357	2.4768
GMP	Nucleotide/Purine metabolism	1.3321	0.41369	0.0054533	2.2633
ATP	Nucleotide	1.5326	0.61596	0.015059	1.8222
Citrulline	Urea Cycle	0.76887	-0.37919	0.016817	1.7743
Glycine	Amino Acid	1.6799	0.7484	0.030133	1.521

Supplemental Table S11. Changed brain metabolites in different metabolic pathways in the fed state by Volcano plots.

Metabolite	Pathway	FC	log2(FC)	P Value	-log10(p)
3-Phosphoglyceric acid	Glycolysis	1.6517	0.72392	0.017088	1.7673
cAMP	Nucleotide/Purine metabolism	1.9955	0.99672	0.031295	1.5045
Glucose	Glycolysis/sugar	2.1654	1.1147	0.028782	1.5409
Glucose 1-phosphate	Glycolysis	1.3453	0.42793	0.01782	1.7491
Glucose 6-phosphate	Glycolysis/PPP	1.4109	0.49657	0.024843	1.6048
Glycerate	Glycolysis	2.4906	1.3165	0.021429	1.669
Hypotaurine	Taurine metabolism	0.43133	-1.2131	0.010304	1.987
Hypoxanthine	Nucleotide/Purine metabolism	0.68879	-0.53787	0.001358	2.867
Kynurenine	Amino Acid metabolism/Trp	1.3636	0.44747	0.047157	1.3265
NAD	Nicotinate and nicotinamide metabolism	0.67971	-0.55701	0.024357	1.6134
Pantothenic acid	Vitamin/CoA	1.3712	0.45546	0.000156	3.8081
PEP	Glycolysis	1.4706	0.55639	0.018721	1.7277
Thiamine	Vitamins	0.59737	-0.74329	0.025238	1.5979
Uracil	Nucleotide/Pyrimidine metabolism	0.74944	-0.41611	0.011571	1.9366

Supplemental Table S12. Changed brain metabolites in different metabolic pathways in the fasted state by Volcano plots.

Metabolite	Pathway	FC	log2(FC)	P Value	-log10(p)
Aconitate	TCA Cycle	1.8474	0.88546	0.023852	1.6225
ADP	Nucleotide/Purine metabolism	0.7174	-0.47915	0.049835	1.3025
ATP	Nucleotide	0.27504	-1.8623	0.013567	1.8675
cGMP	Nucleotide/Purine metabolism	2.0395	1.0282	0.023383	1.6311
CoA	TCA Cycle/Fatty acid metabolism	0.4994	-1.0017	0.040616	1.3913
Cystathionine	Amino Acids/cys	1.6675	0.73766	0.006593	2.1809
Hypotaurine	Taurine metabolism	0.45918	-1.1229	0.049854	1.3023
Methionine	Amino Acid	0.5614	-0.83289	0.023608	1.6269
NADP	Nicotinate and nicotinamide metabolism	0.41096	-1.2829	0.028343	1.5475
Nicotinamide riboside	Nicotinate and nicotinamide metabolism	0.68593	-0.54387	0.045193	1.3449
Oxidized glutathione	Amino acid metabolism	0.45622	-1.1322	0.041305	1.384
Pantothenic acid	Vitamin/CoA	1.474	0.55969	0.000472	3.3258
SAM	Amino Acid/pro and arginine	0.4397	-1.1854	0.007834	2.106
Tyrosine	Amino Acid	0.5899	-0.76147	0.004907	2.3092
Xanthine	Nucleotide	0.66431	-0.59007	0.007764	2.1099

Supplemental Table S13. Changed brain metabolites in different metabolic pathways in the fasted state by Volcano plots.

Metabolite	Pathway	FC	log2(FC)	P Value	-log10(p)
Aconitate	TCA Cycle	1.8474	0.88546	0.023852	1.6225
ADP	Nucleotide/Purine metabolism	0.7174	-0.47915	0.049835	1.3025
ATP	Nucleotide	0.27504	-1.8623	0.013567	1.8675
cGMP	Nucleotide/Purine metabolism	2.0395	1.0282	0.023383	1.6311
CoA	TCA Cycle/Fatty acid metabolism	0.4994	-1.0017	0.040616	1.3913
Cystathionine	Amino Acids/cys	1.6675	0.73766	0.006593	2.1809
Hypotaurine	Taurine metabolism	0.45918	-1.1229	0.049854	1.3023
Methionine	Amino Acid	0.5614	-0.83289	0.023608	1.6269
NADP	Nicotinate and nicotinamide metabolism	0.41096	-1.2829	0.028343	1.5475
Nicotinamide riboside	Nicotinate and nicotinamide metabolism	0.68593	-0.54387	0.045193	1.3449
Oxidized glutathione	Amino acid metabolism	0.45622	-1.1322	0.041305	1.384
Pantothenic acid	Vitamin/CoA	1.474	0.55969	0.000472	3.3258
SAM	Amino Acid/pro and arginine	0.4397	-1.1854	0.007834	2.106
Tyrosine	Amino Acid	0.5899	-0.76147	0.004907	2.3092
Xanthine	Nucleotide	0.66431	-0.59007	0.007764	2.1099

Supplemental Table S14. Changed brain metabolites in different metabolic pathways in fed vs. fasted male mice by Volcano plots.

Metabolite	Pathway	FC	log2(FC)	P Value	-log10(p)
2-Methylbutyroylcarnitine	AcylCarnitine/Amino acid metabolism	1.4493	0.53532	0.0036333	2.4397
Aminoadipic acid	Amino Acid metabolism/Lysine	1.3228	0.40356	0.0045172	2.3451
Urate	Nucleotide/Purine metabolism	1.3555	0.43885	0.0057283	2.242
Trigonelline	Nicotinate and nicotinamide metabolism	0.62827	-0.67054	0.0068483	2.1644
3-Hydroxybutyric acid	Lipid/Ketone bodies	4.239	2.0837	0.014167	1.8487
N1-Methylnicotinamide	NAD Metabolism	1.3323	0.41389	0.019394	1.7123
G3P	Glycolysis	0.60539	-0.72407	0.020246	1.6937
NADP	Nicotinate and nicotinamide metabolism	0.60551	-0.72378	0.027281	1.5641
UTP	Nucleotide/pyrimidine	0.46316	-1.1104	0.034957	1.4565

Supplemental Table S15. Changed brain metabolites in different metabolic pathways in fed vs. fasted female mice by Volcano plots.

Metabolite	Pathway	FC	log2(FC)	P Value	-log10(p)
3-Hydroxybutyric acid	Lipid/Ketone bodies	3.951	1.9822	1.17E-07	6.9321
Isoleucine	Amino Acid	1.3255	0.40648	3.78E-05	4.4228
Tyrosine	Amino Acid	0.47872	-1.0627	0.0002374	3.6246
Trigonelline	Nicotinate and nicotinamide metabolism	0.51904	-0.94609	0.0002443	3.6121
2-Methylbutyroylcarnitine	AcylCarnitine/Amino acid metabolism	1.398	0.48333	0.0011989	2.9212
Lactate	Glycolysis	0.76208	-0.39199	0.0013095	2.8829
Methionine	Amino Acid	0.44833	-1.1574	0.0016478	2.7831
Kynurenine	Amino Acid metabolism/Trp	1.6713	0.74096	0.0029291	2.5333
Cystathionine	Amino Acids/cys	1.7148	0.77801	0.0033708	2.4723
DHAP	Glycolysis	0.33395	-1.5823	0.005669	2.2465
Valine	Amino Acid	1.5442	0.62683	0.0057904	2.2373
cGMP	Nucleotide/Purine metabolism	2.1808	1.1248	0.0075014	2.1249
NADH	Nicotinate and nicotinamide metabolism	0.58112	-0.7831	0.0099077	2.004
PEP	Glycolysis	0.56546	-0.8225	0.010287	1.9877
Aconitate	TCA Cycle	1.8577	0.89349	0.011694	1.932
L-Arginine	Amino Acid/Urea cycle	0.72455	-0.46484	0.012296	1.9102
Oxidized glutathione	Amino acid metabolism	0.42457	-1.2359	0.012386	1.9071
Glucose 1-phosphate	Glycolysis	0.67156	-0.57441	0.015201	1.8181
Lysine	Amino Acid	0.76242	-0.39133	0.015609	1.8066
3-Phosphoglyceric acid	Glycolysis	0.54293	-0.88117	0.018662	1.729
CDP	Nucleotide/Pyrimidine metabolism	0.40885	-1.2903	0.019274	1.715
UTP	Nucleotide/pyrimidine	0.32129	-1.6381	0.022425	1.6493
ATP	Nucleotide	0.21247	-2.2347	0.023385	1.6311
ADP	Nucleotide/Purine metabolism	0.67141	-0.57474	0.024548	1.61
Butyrylcarnitine	Lipid metabolism/Fatty Acid metabolism	1.4806	0.56619	0.02763	1.5586
Heptadecanoic acid	Lipid metabolism/Fatty Acid metabolism	1.3813	0.46602	0.028489	1.5453
NADP	Nicotinate and nicotinamide metabolism	0.36044	-1.4722	0.029498	1.5302

Nicotinamide Riboside	Nicotinate and nicotinamide metabolism	0.68868	-0.53809	0.03521	1.4533
Ornithine	Amino acid/Urea cyle	0.7114	-0.49127	0.035326	1.4519
Isobutyrylcarnitine	Lipid metabolism/Fatty Acid metabolism	1.4548	0.54086	0.040416	1.3934
Glucose	Glycolysis/sugar	0.52179	-0.93845	0.041703	1.3798
Glucose 6-phosphate	Glycolysis/PPP	0.71928	-0.47538	0.047523	1.3231

Supplemental Table S16. Changed plasma metabolites in different metabolic pathways in fed vs. fasted male mice by Volcano plots.

**Metabolite**	**Pathway**	**FC**	**log2(FC)**	**P Value**	**-log10(p)**
Aminoadipic acid	Amino Acid metabolism/Lysine	1.9943	0.99588	0.0009097	3.0411
Glucose	Glycolysis/sugar	0.44742	-1.1603	0.0024337	2.6137
Hypotaurine	Taurine metabolism	0.35	-1.5146	0.0058226	2.2349
Trigonelline	Nicotinate and nicotinamide metabolism	0.20947	-2.2552	0.0065327	2.1849
Cis-aconitic acid	TCA Cycle	0.22744	-2.1364	0.0090333	2.0442
IPP	mevalonate pathway	0.52134	-0.93971	0.009865	2.0059
CDP	Nucleotide/Pyrimidine metabolism	0.48945	-1.0308	0.019984	1.6993
Alanine	Amino Acid	0.64641	-0.62948	0.022802	1.642
3-Hydroxybutyric acid	Lipid/Ketone bodies	7.4789	2.9028	0.024978	1.6024
Lysine	Amino Acid	0.59674	-0.74482	0.027688	1.5577
Cytidine	Nucleotide	1.3384	0.42056	0.04725	1.3256

Supplemental Table S17. Changed plasma metabolites in different metabolic pathways in fed vs. fasted male mice by Volcano plots.

Metabolite	Pathway	FC	log2(FC)	P Value	-log10(p)
Trigonelline	Nicotinate and nicotinamide metabolism	0.15111	-2.7264	8.81E-05	4.0548
Glucose	Glycolysis/sugar	0.50091	-0.99739	0.002212	2.6552
Succinate	TCA Cycle	0.76015	-0.39564	0.0048231	2.3167
Cis-aconitic acid	TCA Cycle	0.44545	-1.1667	0.0066273	2.1787
Lactate	Glycolysis	0.7446	-0.42546	0.018613	1.7302
Lysine	Amino Acid	0.60461	-0.72593	0.020769	1.6826
Betaine	Amino Acid/gly,ser, thr metabolism	0.65289	-0.61509	0.024545	1.61
Glutamate	Amino Acid	0.35916	-1.4773	0.02569	1.5902
Aspartate	Amino Acid	0.40158	-1.3162	0.029626	1.5283
3-Phosphoglyceric acid	Glycolysis	0.36667	-1.4475	0.029718	1.527
Butyrylcarnitine	Lipid metabolism/Fatty Acid metabolism	0.4669	-1.0988	0.03168	1.4992
3-Hydroxybutyric acid	Lipid/Ketone bodies	4.5737	2.1934	0.034554	1.4615
Cysteine	Amino Acid	0.3725	-1.4247	0.039399	1.4045
Thiamine	Vitamins	0.66342	-0.59201	0.04269	1.3697
Ornithine	Amino acid/Urea cyle	0.41608	-1.2651	0.043573	1.3608
Isobutyrylcarnitine	Lipid metabolism/Fatty Acid metabolism	0.51287	-0.96333	0.04642	1.3333
Propionylcarnitine	Acylcarnitine/Fatty Acid metabolism	0.43513	-1.2005	0.046526	1.3323
